# Molecular epidemiology of hepatitis D virus circulating in Southwestern Nigeria

**DOI:** 10.1186/s12985-016-0514-6

**Published:** 2016-04-05

**Authors:** Oluyinka Oladele Opaleye, Oluwatoyin Margaret Japhet, Olubusuyi Moses Adewumi, Ewean Chukwuma Omoruyi, Olusola Anuoluwapo Akanbi, Adeolu Sunday Oluremi, Bo Wang, Hoang van Tong, Thirumalaisamy P. Velavan, C.-Thomas Bock

**Affiliations:** Department of Medical Microbiology and Parasitology, Ladoke Akintola University of Technology, Ogbomoso, Nigeria; Department of Microbiology, Obafemi Awolowo University, Ile Ife, Nigeria; Department of Virology, College of Medicine, University of Ibadan, Ibadan, Nigeria; Institute of Child Health, College of Medicine, University of Ibadan, Ibadan, Nigeria; Department of Infectious Diseases, Division of Viral Gastroenteritis and Hepatitis Pathogens and Enteroviruses, Robert Koch Institute, Seestr. 10, D-13353 Berlin, Germany; Institute of Tropical Medicine, University of Tuebingen, Tuebingen, Germany

**Keywords:** Hepatitis D virus, HDV genotype, Molecular epidemiology, HBV infection, Nigeria

## Abstract

**Background:**

Hepatitis B virus (HBV) and hepatitis D virus (HDV) infections are major public health problems in sub-Saharan Africa. Whereas it is known that HBV infection is endemic in Nigeria, there is only little data about HDV prevalence available. Here, we assessed the HDV seroprevalence and determined the HDV and HBV genotypes distribution among HBsAg positive individuals in Southwestern Nigeria.

**Methods:**

This cross-sectional study involved 188 serum samples from HBsAg positive outpatients recruited at four tertiary hospitals in Southwestern Nigeria. Anti-HDV antibodies were detected by ELISA while HDV-RNA was detected by RT-PCR. Sequencing followed by phylogenetic analyses and HBV genotype-specific PCR were used to characterize HDV and HBV genotypes, respectively.

**Results:**

Out of 188 HBsAg positive serum samples, 17 (9 %) showed detectable HDV-RNA. Anti-HDV antibodies test was possible from 103 samples and were observed in 4.9 % (5/103) patients. There was no significant difference in HDV prevalence between four main cities across the country. 64.7 % of HDV-RNA positive samples were from males and 35.3 % from females (*P* < 0.05). No significant associations were observed with regard to HDV seroprevalence and available demographic factors. Phylogenetic analyses demonstrated a predominance of HDV genotype 1 and HBV genotype E among the HDV-RNA/HBsAg positive patients.

**Conclusions:**

In conclusion, our study showed a high prevalence of HDV infection in HBsAg carriers and the predominance of HDV genotype 1 infection in Nigerian HBV endemic region. The findings contribute to a better understanding of the relevance of HDV/HBV co-infection and circulating genotypes.

## Background

Hepatitis D virus (HDV) is a defective RNA virus presenting similarities to some plant viroids, which requires hepatitis B virus (HBV) as a helper virus for its propagation. Among 240 million chronic HBV carriers reported worldwide [1], approx. 15 to 20 million individuals are also infected with HDV [2–4]. In Africa, of the estimated 65 million chronic HBV carriers, about one fourth of HBsAg-positive individuals show dual-infection with HDV [4]. Chronic HBV/HDV co-infection can lead more often to severe liver diseases, like fulminant hepatitis, if compared to HBV mono-infection [5], whereas HBV/HDV super-infection is associated with chronic infection among 90 % of the virus carriers [3].

HDV is a spherical hybrid particle of ~36 nm in diameter, composed of an outer coat containing hepatitis B surface antigens (HBsAg) and host lipids. The inner nucleocapsid consists of small and large hepatitis D delta antigens (sHDAg and LHDAg) and a single-stranded, circular RNA molecule of ~1.7 kb [6, 7]. The unique open reading frame of the HDV genome encodes the delta antigens. sHDAg is required for HDV genome synthesis while LHDAg inhibits HDV-RNA synthesis and is essential for HDV particle formation [8]. Currently, eight HDV genotypes (HDV1-8) have been described with variable geographical distribution [9–11]. HDV1 is the most common genotype that distributes globally [9]. HDV2 is prevalent in Asia while HDV3 is detectable in South America. In sub-Saharan countries, the prevalence of HDV1, HDV5, HDV6, HDV7 and HDV8 has been reported previously [4, 12, 13]. Clinically, HDV1 infection is associated with both severe and mild liver disease, whereas HDV2 and HDV3 are associated with mild clinical course and outbreaks of severe fulminant hepatitis, respectively [14]. However, there is limited information on the clinical course of the other five HDV genotypes.

The HDV prevalence has been reported in various parts of the world. In Africa, the anti-HDV antibody prevalence in HBsAg carriers was reported only in Cameroon (17.6 %) and Gabon (15.6 % to 70.6 %) [13, 15, 16]. Recently, HDV prevalence in sub-Saharan Africa was estimated from 1.3 % to 50 % [4]. Although HBV is endemic in Nigeria, data on HDV seroprevalence are limited. A previous study showed that HDV antigen was detectable in 6.5 % of patients with chronic hepatitis B in Southwest Nigeria [17]. In addition, another study reported an anti-HDV prevalence of 12.5 % in 96 HBsAg positive patients [18]. Moreover, a recent study showed that HDV1 prevails with 53.3 % in Southwestern Nigeria followed by the HDV5 (33.3 %) and HDV6 (13.3 %), which were more restricted to the northern part of Nigeria [4].

Early childhood transmission is considered to be the most important route of HBV infection in high endemic areas therefore HDV super-infections contribute considerably to the high burden of chronic liver disease [12]. Although the HBV vaccination program into the routine children immunization schedule has been introduced in 2004 [19], HBV infection still remains a major public health problem in Nigeria with 10–15 % of the population positive for HBsAg [20]. Molecular epidemiology studies of HDV prevalence and determination of circulating HBV and HDV genotypes in HBV endemic regions of Africa are needed to assess and improve the control measures of HBV/HDV co-infection. In this study, we therefore investigated the HDV seroprevalence and circulation of HBV and HDV genotypes in HBsAg positive serum samples from Southwestern Nigeria.

## Methods

### Study area and serum sample collection

One hundred and eighty-eight serum samples obtained from HBsAg positive outpatients were included in this cross-sectional study conducted between June and September 2014 in four tertiary hospitals in southwestern Nigeria. The four tertiary hospitals were Ladoke Akintola University Teaching Hospital in Osogbo, Ladoke Akintola University Teaching Hospital Ogbomoso, Obafemi Awolowo Teaching hospital Ile-Ife, and University College Teaching Hospital Ibadan. Of the 188 samples, 123 were from Ibadan, 26 from Ogbomoso, 24 from Osogbo, and 15 from Ife. The outpatients came to the hospital for various complaints and routine examination. Therefore, no patient with severe liver disease was recruited. No information about treatment history was available from these outpatients. Due to small volume of the samples only 103 samples could be analysed for the presence of anti-HDV antibodies.

### Ethics

Ethical approval was obtained from the Ladoke Akintola University Teaching hospital ethical committee. Informed written consent was sought and obtained from each of the participants in this study.

### Serology of HBV and HDV

All samples had been tested and confirmed for HBsAg positivity using Rapid Kit (Micropoint Rapid Diagnostic, Micropoint Bioscience, Switzerland). Relative sensitivity and specificity were >99 % and 97.0 % respectively, with accuracy of 98.5 %. HBsAg negative serum samples were excluded from the study. However, anti-HIV and anti-HCV analyses have not been done in this study. Total anti-HDV antibodies (IgG and IgM) were detected using ELISA (ETI-AB-DELTAK-2, Dia-Sorin, Italy). All serological tests were performed following the manufacturer’s instructions.

### Detection of HDV-RNA

Nucleic acid was extracted from patient sera using High Pure Viral Nucleic Acid Kit (Roche, Grenzach-Wyhlen, Germany) following the manufacturer’s instruction and stored until use in aliquots at -80 °C. We employed HDV-specific nested RT-PCR approach for HDV detection using primers designed for amplification of a highly conserved region of the HDV genome (LHDAg region, nucleotide 888 to 1122). The first round of RT-PCR was performed using a one-step RT-PCR kit (QIAgen, Hilden, Germany) and primer pair of HDV-04 and HDV-05 [21]. The primer pair HDV-06 and HDV-07 was used for nested PCR. Primer design and localization in the HDV genome has been previously described [21]. cDNA synthesis and pre-denaturation was performed at 50 °C for 30 min followed by 95 °C for 15 min. PCR amplification was for 35 cycles including: denaturation at 94 °C for 30 s, annealing at 56 °C for 30 s, extending at 72 °C for 45 s, followed by a final extension for 10 min at 72 °C. The nested PCR was initiated by a denaturation step at 95 °C for 2 min and 29 cycles at 94 °C for 30 s, 58 °C for 30 s, and 72 °C for 45 s, followed by a final extension for 5 min at 72 °C. 5 μl of each PCR product was analysed by 1.5 % agarose gel electrophoresis. A positive HDV PCR product was described by a band size of 235 bp (first PCR 323 bp) on the agarose gel. In order to avoid cross contamination, sample processing (DNA/RNA extraction, template preparation, and master mix preparation) and PCR was performed in separate laboratory rooms, which are all certified for molecular diagnostics using standard precautions.

### HDV genotyping and phylogenetic analysis

HDV genotyping was performed by using direct sequencing. HDV positive PCR fragments were extracted from agarose gelelectrophoresis and purified using a gel extraction kit (Qiagen MinElute gel extraction kit, Hilden, Germany) according to the manufacturer’s instruction. Sequencing reactions were performed using 1–5 μl purified PCR products, 1 μl BigDye reaction mix (Life Technologies, Applied Biosystems, Darmstadt, Germany) and 0.5 μM of the primers HDV-06 and HDV-07. Sequencing results were analyzed using BioEdit 9.7 software (http://www.mbio.ncsu.edu/BioEdit/bioedit.html) and Geneious Pro (Version 5.5.7, Biomatters Ltd, Auckland, New Zealand, http://www.geneious.com). The phylogenetic tree reconstruction and the mean value of genetic diversity of DNA sequences were carried out using MEGA 5 software [22]. The evolutionary history was inferred using the Neighbor-Joining method. The evolutionary distances were computed using the Maximum Composite Likelihood method. For alignment and HDV genotyping, eight prototype HDV-genotype sequences retrieved from the NCBI Gene bank were used (HDV-genotype 1: X77627, M92448, AB118848, AJ000558, X85253, AY633627, AF098261; HDV-genotype 2: AJ309880, X60193, U19598, AF104624; HDV-genotype 3: AB037948; HDV genotype 4: AF209859; HDV-genotype 5: AM183326; HDV genotype 6: AM183329; HDV-genotype 7: AM183333; HDV genotype 8: AX741169).

### HBV genotyping

HBV-DNA was amplified by HBV genotype-specific nested PCR using generic outer primers (P1 and S1-2), followed by duplex primer mixtures containing type-specific inner primers as described elsewhere [23].

### Statistical analysis

Statistical analysis was performed using SPSS version 15 (IBM Corporation, Armonk, NY, USA). Categorical data were compared by Fisher’s exact test. Non-parametric data were compared by using the Chi-square test, with a 2-tailed *P* value <0.05 considered to be statistically significant.

## Results

### Sample characteristics and HDV prevalence

**Fig. 1:**
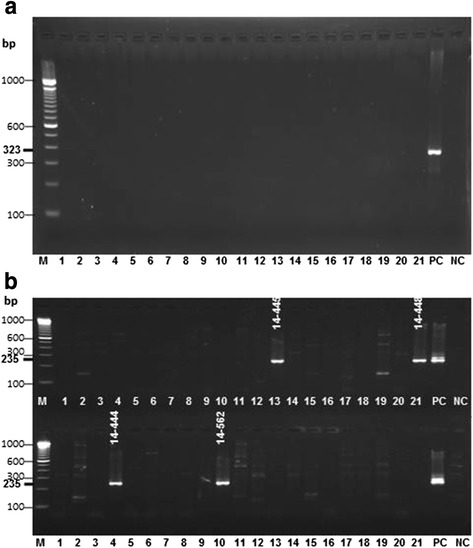
Representative agarose gel electrophoreses of amplified HDV-products. **a** HDV specific nRT-PCRs showing results of the first PCR round (HDV-specific amplicon of 323 bp) and **b** nested PCR with an HDV-specific amplicon of 235 bp. Lanes 1-21 correspond to PCR results from serum samples. HDV-positive samples with correct size in (**b**) are denoted with the patient number, e.g., 14-445 (see (**b**) lane 13 and 21; upper lanes, and lane 4 and 10, lower lanes). PC is the HDV-positive control (acc. No. M21012, [27]); NC is the negative control. Lane M shows a 100 bp DNA ladder, base pairs (bp) of the marker are denoted at the left

Of the 188 HBsAg positive serum samples from outpatients recruited at four tertiary hospitals in Southwestern Nigeria, 82 (43.6 %) were from females and 106 (56.4 %) were from males. The mean age of all the HBsAg positive patients was 32 ± 10.8 years, and there was no significant difference between males (32.2 ± 11.4 years) and females (31.8 ± 10.5 years). Due to the low volume of serum samples available, only 103 of the 188 HBsAg positive samples could be examined for prevalence of anti-HDV antibodies; however, HDV genome detection was possible from all 188 samples. Of 103 samples, five (4.9 %) were positive for anti-HDV antibodies (IgM and IgG). Unexpectedly, none of the anti-HDV antibodies positive samples were HDV-RNA positive. 17 of the 188 HBsAg-positive serum samples demonstrated detectable HDV-RNA given a HDV prevalence of 9 % in our study population (Table 1, Fig. 1).

**Table 1:** Patient data, detection of HBsAg and anti-HDAg in Nigerian patient samples

Patient	Age (years)	Gender (f/m)	Region/City	HBsAg (+/-)	anti-HDV λ450_620nm	HDV-RNA (+/-)	HDV genotype
14-366	45	f	Osogbo	+	n.d.	+	1
14-374	n.d.	n.d.	Ibadan	+	n.d.	+	1
14-375	n.d.	n.d.	Ibadan	+	n.d.	+	1
14-386	30	m	Ibadan	+	0.021	-	
14-387	34	f	Ibadan	+	n.d.	+	1
14-388	38	m	Ibadan	+	n.d.	+	1
14-389	n.d.	m	Ibadan	+	n.d.	+	1
14-392	18	f	Ibadan	+	0.884	-	
14-403	25	m	Ibadan	+	n.d.	+	1
14-406	24	f	Ibadan	+	n.d.	+	1
14-408	14	m	Ibadan	+	n.d.	+	1
14-415	24	m	Ibadan	+	1.016	-	
14-420	14	m	Ibadan	+	0.803	-	
14-425	33	f	Ibadan	+	0.701	-	
14-426	68	f	Ibadan	+	n.d.	+	1
14-432	n.d.	m	Ibadan	+	1.044	-	
14-444	n.d.	f	Ibadan	+	n.d.	+	1
14-445	34	m	Ife	+	n.d.	+	1
14-448	30	m	Ife	+	n.d.	+	1
14-460	46	f	Ibadan	+	0.277	-	
14-472	19	m	Ibadan	+	n.d.	+	1
14-495	49	m	Ibadan	+	n.d.	+	1
14-496	34	m	Ibadan	+	n.d.	+	1
14-509	32	m	Ibadan	+	1.128	-	
14-562	n.d.	n.d.	Osogbo	+	n.d.	+	1

Patient numbers are indicated as 14-XXX; the region/cities are located in south-western Nigeria within 200 km; OD (λ 450_620 nm); OD of >1.2 is not reactive, OD of 0.9-1.2 is borderline reactive, OD <0.9 is reactive. n.d. is not defined or data not available

Determination of gender affinity showed that 11 of the 17 (64.7 %) HDV-RNA positive serum samples were from males and six (35.3 %) were from females (*P* = 0.6). A more detailed analysis of the regional distribution in Southwestern Nigeria showed that HDV-RNA detection in HBsAg-positive individuals is more frequent in Ife (2/15; 13.3 %) than in any of the three remaining cities including Osogbo (2/24; 8.3 %), Ibadan (12/123; 9.8 %), and Ogbomoso (1/26; 3.8 %). However, the difference was not statistically significant (P > 0.05). Analysis of the age dependency of HDV-RNA detection showed that patients within the age range of 31–40 years had highest prevalence of HDV-RNA with 7/17 (41.2 %), followed by the age group of 21–30 years with 3/17 (17.6 %). The patients with age range of 11–20 years, 41–50 years and ≥51 years had similar prevalence of HDV-RNA with 2/17 (11.8 %). As expected, the lowest HDV-RNA prevalence was found within the age group of 0–10 years with 1/17 (5.9 %).

### HDV and HBV genotype distribution in the Nigerian study population

**Fig. 2:**
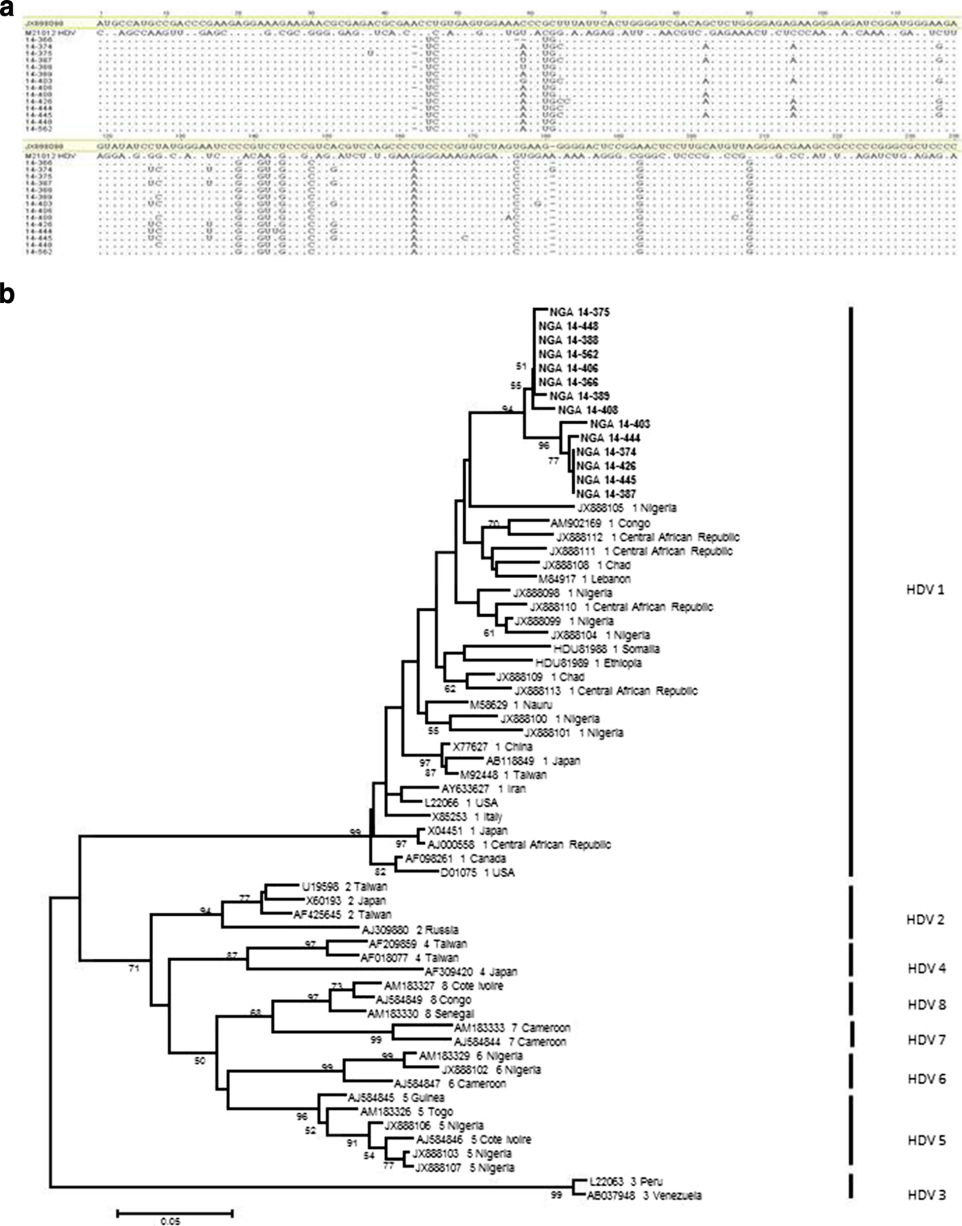
**a** Representative HDV sequences of the HBsAg-positive patients aligned with the Nigerian HDV reference sequence JX888098. HDV sequences spanning the region from nt 887 to nt 1127 showing patient-specific HDV isolates. Notably, HDV reference sequences of pSVL-D3 (Acc. No. M21012; positive control of the nRT-PCR) has been included to show that there is no cross-contamination. **b** Phylogenetic analysis inferred from distance analysis (Kimura 2 parameters model) using neighbor-joining bootstrap 1000 replicate reconstruction from HDV-sequences (nt 888 to nt 1122) of the Nigerian HDV isolates (highlighted in boldface and designated as NGA and numbers, e.g NGA 14-375) and the corresponding region of reference sequences showing that the Nigerian HDV isolates clustered in the HDV genotype 1 branch. The Nigerian HDV sequences were compared to HDV reference sequences gathering the 8 HDV genotypes (HDV1 to HDV8) which are denoted at the right in brackets (accession number, genotype, and geographic area are denoted in the figure). The numbers at the nodes indicate bootstrapping values. The bar represents nucleotide substitutions per position. HDV sequences of the patient isolates are available at NCBI Genebank database (Acc. No. KU844264 to KU844277)

In order to determine the circulation of HDV genotypes in Southwestern Nigeria, sequence data for nucleotide 888-1122 of HDV-RNA (LHDAg region) were obtained for 14 of the 17 HDV RNA-positive samples. The obtained HDV sequences were aligned using BioEdit, Geneious v. 8.1, and phylogenetic analysis was performed with prototype HDV sequences obtained from the NCBI GenBank database. The phylogenetic analysis of the 14 HDV isolates showed that all isolates clustered within the HDV genotype 1 clade (Fig. 2). HDV sequences are available at the NCBI GenBank database (Acc. No. KU844264 to KU844277). Additionally, in order to determine the HBV genotype in the HDV-RNA positive serum samples, HBV genotype-specific multiplex PCR was performed and the result showed that HBV genotype E prevails in all 17 HDV-RNA positive serum samples.

## Discussion

Although HBV vaccination under the children immunization schedule has been available in Nigeria since 2004, HBV infection still remains a serious public health issue in this country with a prevalence of 10–15 % in the population [20, 24]. Among the factors that shape the clinical course of HBV, co-infection with other viruses such as HCV, HIV and HDV is one major risk factor. In this study, we showed a high HDV infection prevalence of 9 % in the Nigerian study population positive with HBsAg and the HDV1 was frequently circulating in Southwestern Nigeria.

Co-infection of hepatitis viruses is common because these viruses share the same route of transmission. HDV infection requires the presence of the HBV surface proteins (HBsAg) to assemble the infectious virus [6]. Therefore, HDV infection occurs regularly where HBV infection is endemic. Data concerning the prevalence of HDV infection in Nigeria as well as in other sub-Saharan African countries are limited. Nevertheless, a recent study showed that among HBsAg positive serum samples in endemic regions, approximately 10 % were positive for anti-HDV antibodies and/or HDV-RNA ranging from <2 % detected in pregnant women in Burkina Faso to approx. 50 % of liver disease patients in Central African Republic [4]. Our study on HBsAg positive individuals from four cities in Southwestern Nigeria showed that 9 % were HDV-RNA positive and therefore representing active HDV infection. Only 4.9 % of patients were anti-HDV antibody positive, which is significantly lower compared to another recent study showing 12.5 % anti-HDV antibody positivity [18]. However, asymptomatic infected patients of this study showed an anti-HDV prevalence of 4.3 % [18]. This value is in good agreement to our finding of 4.9 % analysing only asymptomatic individuals. In addition, another previous study reported a HDV antigen prevalence of 6.5 % in Nigerian HBsAg positive patients [17].

A limitation of our study is that we had not enough sample volume from each HBsAg-positive patient at disposal. Therefore, we could test only 103 of 188 serum samples for anti-HDAg antibodies. However, none of the anti-HDAg positive samples was HDV-RNA-positive and on the other hand none of the HDV-RNA positive samples tested positive for anti-HDAg (Table **1**). Accordingly, we can only speculate about acute or chronic HDV infection of the patients. However, it can be hypothesized that the anti-HDAg positive patients without HDV-RNA detection were likely having chronic infection.

The results of our study revealed a high prevalence of HDV infection, which may contribute to significant morbidity and mortality in HBV-related liver diseases in Nigeria. In addition, although not statistically significant, analysis of gender affinity to HDV infection in our study indicated that male gender is predominantly affected with HDV (64.7 %) whereas female showed only 35.3 % of the total HDV positive cohort. This is not unexpected and in concordance with a recent report showing that more than 75 % of HDV infected patients were males [21].

At least eight HDV genotypes have been identified and HDV1 is the most common and is distributed throughout the world [25]. The predominance of HDV1 has been also described for sub-Sahara Africa [11, 25]. In this regard, our result showed that HDV1 was predominant in Southwestern Nigeria and no African-specific HDV5 to HDV8 were identified. However, HDV5 and HDV6 prevalence has been reported in the northern part of Nigeria [4]. Therefore, the samples from other parts in Nigeria are needed to characterize the predominance of circulating HDV genotypes in this country. Furthermore, HBV genotype E is the predominant HBV genotype detectable in Nigeria [26]. In agreement with this report, HBV genotype analyses of the HDV positive samples of our study cohort revealed that HBV genotype E predominates in Southwestern Nigeria. A close co-evolution of HDV1 and HBV genotype E seems unlikely, since HDV1 prevails worldwide whereas HBV genotype E is largely confined to Africa and is thought to have a short evolutionary history [4].

## Conclusions

In conclusion, our study provides important information in the HDV infection prevalence and molecular epidemiology of HBV and HDV genotypes circulating in endemic regions such as Southern Nigeria, which significantly contributes to improve the control measures of these hepatitis viruses. Future surveillance studies of HBV and HDV infections are therefore of public health importance not only for Nigeria but also for endemic regions in sub-Sahara Africa.
